# The role of the microbiota in the management of intensive care patients

**DOI:** 10.1186/s13613-021-00976-5

**Published:** 2022-01-05

**Authors:** Piotr Szychowiak, Khanh Villageois-Tran, Juliette Patrier, Jean-François Timsit, Étienne Ruppé

**Affiliations:** 1 Université de Paris, IAME, INSERM, 75018 Paris, France; 2 Service de Médecine Intensive-Réanimation, Centre Hospitalier Régional Universitaire de Tours, 37000 Tours, France; 3 Laboratoire de Bactériologie, AP-HP, Hôpital Beaujon, 92110 Paris, France; 4 Service de Réanimation Médicale Et Infectieuse, AP-HP, Hôpital Bichat, 75018 Paris, France; 5 Laboratoire de Bactériologie, AP-HP, Hôpital Bichat-Claude Bernard, 46 rue Henri Huchard, 75018 Paris, France

**Keywords:** Microbiota, Dysbiosis, Fecal microbiota transplantation, Probiotics, Intensive care, *C. difficile*, Multidrug-resistant bacteria

## Abstract

The composition of the gut microbiota is highly dynamic and changes according to various conditions. The gut microbiota mainly includes difficult-to-cultivate anaerobic bacteria, hence knowledge about its composition has significantly arisen from culture-independent methods based on next-generation sequencing (NGS) such as 16S profiling and shotgun metagenomics. The gut microbiota of patients hospitalized in intensive care units (ICU) undergoes many alterations because of critical illness, antibiotics, and other ICU-specific medications. It is then characterized by lower richness and diversity, and dominated by opportunistic pathogens such as *Clostridioides difficile* and multidrug-resistant bacteria. These alterations are associated with an increased risk of infectious complications or death. Specifically, at the time of writing, it appears possible to identify distinct microbiota patterns associated with severity or infectivity in COVID-19 patients, paving the way for the potential use of dysbiosis markers to predict patient outcomes. Correcting the microbiota disturbances to avoid their consequences is now possible. Fecal microbiota transplantation is recommended in recurrent *C. difficile* infections and microbiota-protecting treatments such as antibiotic inactivators are currently being developed. The growing interest in the microbiota and microbiota-associated therapies suggests that the control of the dysbiosis could be a key factor in the management of critically ill patients. The present narrative review aims to provide a synthetic overview of microbiota, from healthy individuals to critically ill patients. After an introduction to the different techniques used for studying the microbiota, we review the determinants involved in the alteration of the microbiota in ICU patients and the latter’s consequences. Last, we assess the means to prevent or correct microbiota alteration.

## Background

In the gut, the microbiota mostly comprised bacteria, but it also harbors archaea, viruses, protozoans, and fungi. The composition of the gut microbiota is unique to each individual in that the gut microbiota of two given individuals consistently show differences in their composition [1]. Nonetheless, it is also highly dynamic and evolves throughout life under the influence of a wide diversity of genetic, environmental, medical, and dietary determinants.

In the intensive care setting, the gut microbiota of patients is submitted to various stresses including antibiotic exposure, modification of gastro-intestinal transit, artificial nutrition or sepsis which may lead to a dysbiosis during hospitalization. Indeed, the gut microbiota in critically ill patients appears to be different from that of healthy subjects, demonstrating markedly lower richness and diversity, and the near replacement of commensal genera by opportunistic pathogens [2]. Recent evidence has shown that dysbiosis in ICU patients might have consequences on survival, stressing that dysbiosis could be considered as an authentic, organ-failure-affecting prognosis along with renal, cardiac, or respiratory failures [3].

The gut microbiota is also the main reservoir for multidrug-resistant bacteria organisms (MDRO). Initially kept at low intestinal concentrations as a consequence of the barrier effect exerted by commensal anaerobic bacteria [4], they may bloom after antibiotic exposure and increase the risk their involvement in further infections. In the present review, we aim at introducing to intensive care practitioners the gut microbiota basics, how its composition is altered during hospitalization in the intensive care setting and the potential consequences for the patient. Finally, we will review the possible interventions for preventing or correcting dysbiosis and discuss how they could be implemented in the intensive care context.

## Methods for studying the gut microbiota (glossary available in Table 1)

**Table 1:** Glossary of terms specific to the microbiota research (alphabetical order)

16S rRNA gene sequencing	A method to analyze bacterial communities by sequencing one or more variable regions of the bacterial 16S rRNA gene by NGS
Abundance	Total number of a given taxa in a sample
Actinobacteria	This phylum is mainly composed by Gram-positive bacteria, but is less abundant in gut microbiota. Notably represented by the *Bifidobacterium* genera, it contains also clinically relevant genera such as *Corynebacterium*, *Nocardia, Actinomyces* or *Mycobacterium*
Alpha-diversity	Refers to the composition within a sample
Bacteroidetes	One of the most represented phyla in gut microbiota. It includes mainly commensals and is composed especially by Gram-negative bacilli such as *Bacteroides*, *Porphyromonas*, or *Prevotella*
Beta-diversity	Refers to the similarity of the composition between samples
Culturomics	Method for analyzing bacterial composition of complex samples such as human gut, based on extensive culture media and atmosphere combinations
Diversity	Describes the number of various bacterial communities and their distribution
Firmicutes	One of the most represented phyla in gut microbiota. It includes mainly Gram-positive bacteria such as genera *Staphylococcus*, *Enterococcus*, *Streptococcus*, or *Lactobacillus*. *Clostridium* genera represent 95% of the Firmicutes phyla in the gut
Next-generation sequencing	Refers to sequencing methods which emerged after the mid-2000s, and which typically yield higher output than Sanger sequencing
Operational taxonomic unit (OTU)	Clusters of sequences sharing a minimal identity (e.g., 97% is commonly used in 16S studies), referring to a taxonomic group. These clusters and the respective number of reads within are an estimation of the abundance of different taxa in samples
Phylum	Taxonomic rank that ranks above class and below kingdom. Classical phyla in gut microbiota studies are Firmicutes, Bacteroidetes, Actinobacteria and Proteobacteria. Firmicutes and Bacteroidetes typically represent 90% of gut microbiota in subjects not exposed to antibiotics
Proteobacteria	Quasi-exclusively composed by Gram-negative bacteria, this phylum includes especially pathogenic genera such as *Escherichia*, *Klebsiella*, *Legionella*, *Pseudomonas*, *Acinetobacter* or *Stenotrophomonas*
Protist	Kingdom including predominantly eukaryotic unicellular microscopic organisms
Richness	Number of different bacterial taxa in a sample
Shotgun sequencing	A method to analyze bacterial communities by sequencing random DNA fragments by NGS
Taxon	Taxonomic group of any rank, such as species, family, or class

The study of the human microbiota is a complex task considering than an estimated 70% of the microbes in the human body have not been cultured yet [5]. Indeed, the extreme susceptibility to oxygen of some intestinal bacteria requires strict oxygen-free culture methods, which is hardly achievable in routine conditions and requires specific equipment typically exclusive to research settings. Thus, the composition of the intestinal microbiota has mostly been unveiled by culture-independent methods such as molecular ones, especially since the 2010s and the rise of next-generation sequencing (NGS).

### Molecular analysis approach

**Fig. 1:**
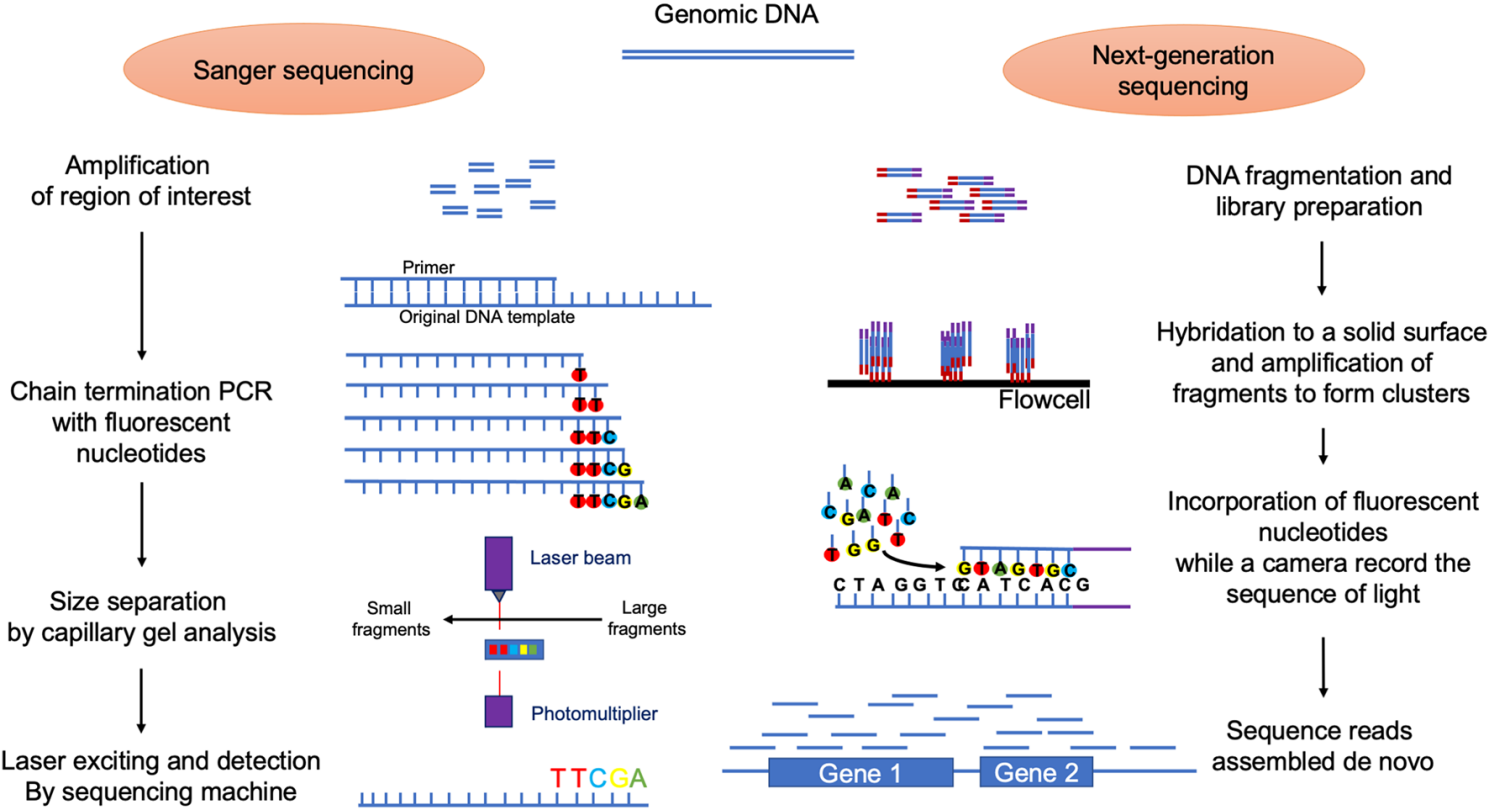
The Sanger sequencing method and next-generation sequencing steps. Sanger sequencing relies on dye-labeled nucleotides that are added to an elongating DNA strand thus determining each base according to the color of the dye. One of the NGS technique allows the specific amplification of an isolated DNA fragment by slide fixation such as in the Illumina chemistry, which is based on-chip amplification in bridge connection that allows simultaneous identification of DNA bases which emit a unique fluorescence signal when they are incorporated into the nucleic acid chain. PCR: polymerase chain reaction. NGS: next-generation sequencing

**Fig. 2:**
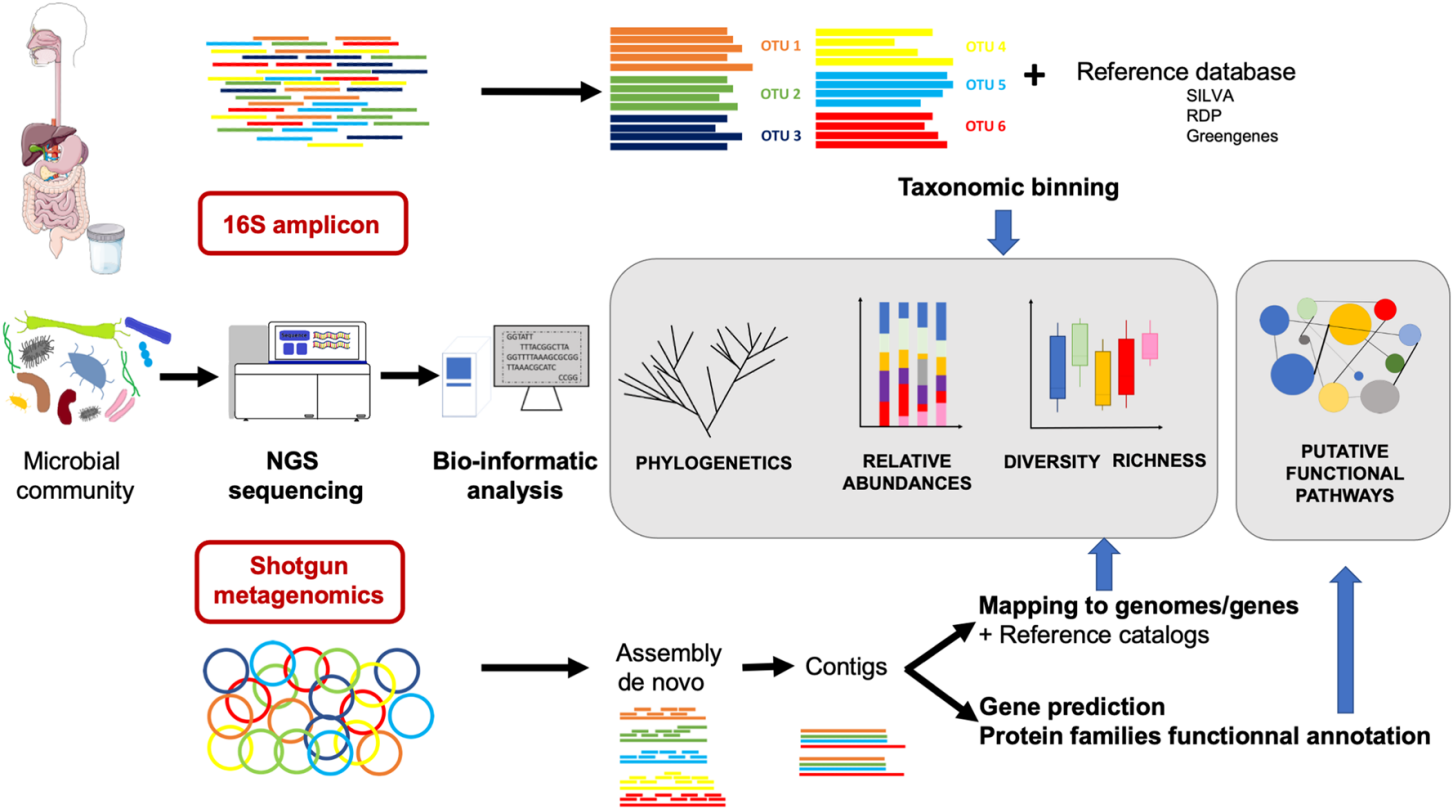
Schematic representation of classically used next-generation sequencing methods. 16S rRNA coding gene amplification and shotgun metagenomics are well-used techniques for microbiota studies. OTU: operational taxonomic unit (an OTU being assumed to be a bacterial genus).NGS: next-generation sequencing

In the mid-2000s, a breakthrough in DNA sequencing occurred with the rise of next-generation sequencing (NGS). The revolution came from two main improvements from the canonical Sanger sequencing: NGS typically yields hundreds of thousands (now billions) of DNA strands in a single experiment and does not require knowledge of the genetic surroundings of the region to be sequenced (Fig. 1). NGS methods have thus been welcomed by researchers as a means of deciphering the microbial composition of our microbiota with two main approaches: 16S profiling and shotgun metagenomics (Fig. 2).

### 16S profiling

**Fig. 3:**
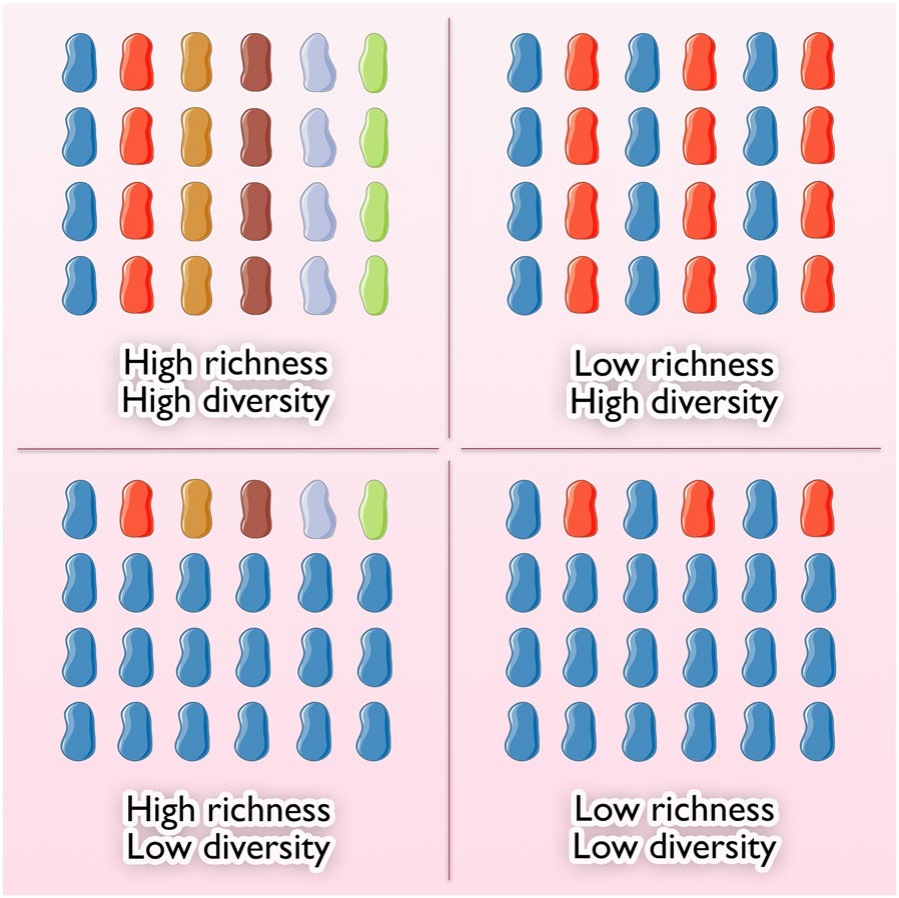
Schematic representation of bacterial richness and diversity concepts. In the figure, each color represents a different bacterial taxon. Richness and diversity can be considered at different levels of taxa, from phylum to species. Richness represents the variety of bacterial communities observed in a specific ecosystem and is greater with the number of different bacterial species found in it. Diversity is related to the preponderance of bacterial communities one over the others, in the ecosystem: when a bacterial taxon is overrepresented in the niche, microbial diversity decrease. Various indices are used to estimate the bacterial diversity. Shannon index estimates the bacterial diversity and increases with the number of different species in an ecosystem. Simpson index increases with the probability that two random species within an ecosystem are the same and is therefore higher when one or more bacterial species are preponderant in an ecosystem. The figure includes material available from Servier Medical Art (https://smart.servier.com) under a Creative Commons license

16S rRNA profiling consists in the amplification (by PCR) and the sequencing (initially by the Sanger protocol, now by NGS) of a targeted ubiquitous microbial DNA, the 16S ribosomal subunit-encoding gene. In a typical output, thousands of short DNA sequences (referred to as reads) are obtained, each being an identifying barcode of the bacterial species to which it belongs. Hence, bioinformatic methods can assign the name of bacteria to every read. From this taxonomic data, the relative abundance of bacterial populations can be assessed and tested for differences according to given parameters such as alpha and beta-diversity indices. Alpha-diversity refers to the number of total taxa within a given sample (richness) and the evenness with which they distribute (Shannon and Simpson indices) (Fig. 3). The beta-diversity refers to the analysis of the composition of different samples in that the distance between samples can be calculated by various methods (e.g., Bray–Curtis, Spearman, UniFrac) and visualized on a scatterplot. The advantage of 16S profiling is the requirement of a limited number of reads (magnitude of thousands) which lowers the cost and the computational needs (in terms of storage and calculation requirements). The limitation is given by the size of the reads which are usually short and may compromise the precise taxonomic assignment of the read down to its species. For instance, a read originating from Staphylococcus aureus (species level) may be recognized as coming from a Staphylococcus spp (genus level), or even from a Staphylococcaceae (family level). In addition, 16S profiling only provides data about the taxonomic composition of a sample, and not about any other data such as the functions of genes. In that, 16S profiling does not help in identifying antibiotic resistance genes, for instance. Last, it does not address non-bacterial members of the microbiota such as yeasts and protists, and suffers from the possible biases intrinsic to the initial PCR step (in that depending on the primers used, some bacterial groups might be promoted at the expense of others).

### Shotgun metagenomics

More complete is “Shotgun Metagenomics” which refers to the sequencing of all the DNA in each sample, whatever its origins. Hence, more information can be retrieved such as the functions of the genes (e.g., metabolic pathways, virulence genes and antibiotic resistance genes). As the taxonomic assignment does not only rely on a small part of the 16S rRNA encoding gene, deeper taxonomic ranks are accessible, and even strains from the same species can be distinguished [6]. However, shotgun sequencing typically requires a high amount of reads (magnitude of millions), thereby increasing cost and computational needs. The same alpha and beta-diversity analyses can be performed with shotgun metagenomics, but not only based on taxonomic data. For instance, the difference in the antibiotic resistance gene composition can be tested between two populations [7].

### Culture methods

Pasteurian microbiology has historically relied on culture techniques that stand on pure isolate culture, thereby narrowing its scope to culturable microorganisms. In the gut microbiota, 10 to 30% of the bacteria are estimated to be culturable with conventional methods such as those used in routine microbiology laboratories [5]. In order to expand the range of culture conditions and thereby that of culturable bacteria, some groups have combined various media and atmosphere conditions to MALDI-TOF (matrix assisted laser desorption ionization—time of flight) mass spectrometry, later designated as “culturomics” [8]. Lagier et al. defined 70 best culture conditions allowing the identification of almost 80% of bacteria present in the gut microbiota. Once identified, pure culture isolates can be sequenced, and their genome can enrich databases used to obtain the taxonomic patterns of samples in NGS data. However, culturomics is significantly labor-intensive and cannot easily be applied to a high number of samples.

## Composition of the gut microbiota in healthy subjects

**Fig. 4:**
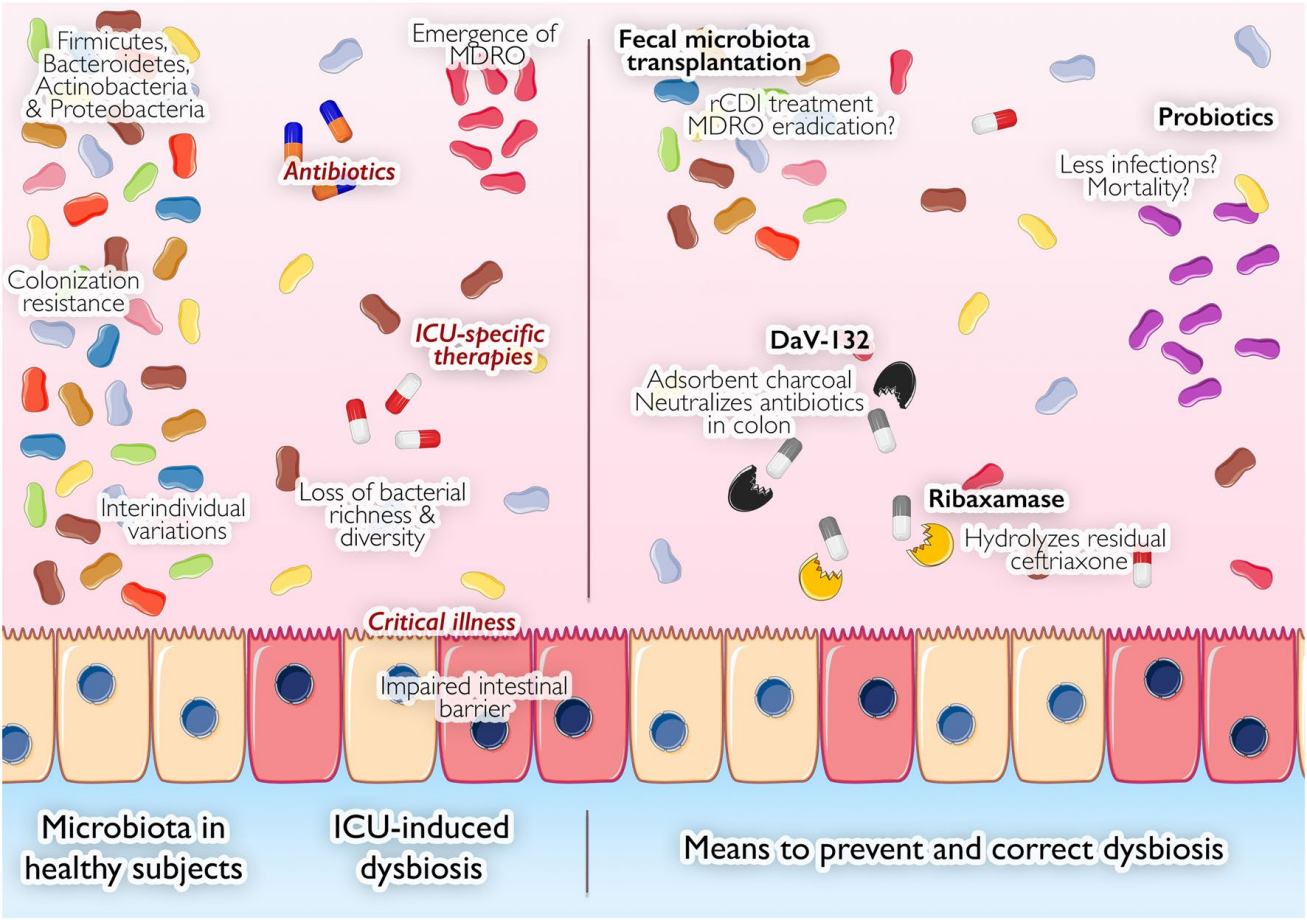
Main microbiota variations in ICU patients and available means to restore dysbiosis. In healthy subjects, microbiota presents important interpersonal variations, but is always composed by four major phyla and one of its important roles is resistance to colonization by exogenous bacteria. In ICU, many factors (rust color) alter the microbiota integrity with numerous consequences. In the right part of the figure, are illustrated the classically described treatments (bold and black letters) playing a role on the microbiota and compensating or preventing alterations. The fecal microbiota transplantation is the most popular microbiota-associated treatment, but other solutions aiming at preserving or restoring the integrity of the microbiota continue to be investigated. *MDRO* multidrug-resistant organism, *ICU *intensive care unit, *rCDI* recurrent *Clostridioides difficile* infection. The figure includes material available from Servier Medical Art (https://smart.servier.com) under a Creative Commons license

Combined data from the MetaHIT [9] and the Human Microbiome Project (HMP) [10–12] consortia have provided the first large characterization of human-associated microorganisms [13]. The vast majority of the gut bacteria distribute into four major phyla: Firmicutes (60–75%), Bacteroidetes (30–40%), Actinobacteria and Proteobacteria [14] (Fig. 4). The phylum Bacteroidetes (including for instance *Bacteroides *spp. and *Prevotella *spp.), is made of Gram-negative anaerobic, non-spore-forming bacteria. Firmicutes is mainly made of Gram-positive obligate or facultative aerobic cocci (e.g., *Enterococcus *spp.) and bacilli (e.g., *Clostridium *spp.). The phylum Actinobacteria, including *Bifidobacterium*, are Gram-positive rod-shaped anaerobic non-motile non-spore-forming bacteria. The phylum Proteobacteria, comprising the notorious enterobacteria (now designated under the Enterobacterales order) are aero-anaerobic facultative Gram-negative rod-shaped non-spore-forming bacteria [15]. Besides bacteria, archaea make up approximately 0.2% of the gut microorganisms [16], gut virome is mainly constituted by bacteriophages, and fungal microbiota (also known as “mycobiota”) contains notably *Candida *spp. and *Saccharomyces *spp., with *Candida albicans* being commensal of the human gut microbiota. The presence and relative abundance of specific microbial communities defining healthy state is difficult to establish for all human beings, and to date, there is no definition of a healthy microbiota, but as many microbiotas as there are healthy subjects.

## Concept of dysbiosis and association with disease

It has been repeatedly demonstrated that the composition of the microbiota of healthy subjects differs from that of patients suffering from a wide array of conditions. An unbalanced state of the microbiota when compared with that of healthy controls is hence referred to as dysbiosis. This disequilibrium is believed to alter the dialogue between the microorganisms and the host’s cells that could contribute to acute or chronic disorders with intestinal and extra-intestinal symptoms. A healthy intestinal microbiota plays a crucial role in host immune regulation. Germ-free mice have an altered immunity compared to healthy mice [17], highlighting the importance of the microbiota in host immune development. These interactions between the microbiota and the metabolites such as short-chain fatty acids (SCFA; e.g., butyrate, acetate, propionate) interact with intestinal receptors and may play an anti-inflammatory role. SCFAs are produced by specific bacterial species and the decrease of these bacteria seems to promote a pro-inflammatory state [18]. Another illustrative example is the gut–brain axis, whose underlying mechanisms are beginning to be studied in greater detail. Gut microbes and the nervous system dialogue via neural, endocrine, and immunological pathways through neuromodulator metabolites (i.e., SCFAs), vitamin B12, neurotransmitters (i.e., serotonin), hormones (i.e., peptide YY) and xenobiotics produced by gut microbes. Hence, gut microorganisms may influence cognitive function and behavior (such as anxiety, depression, autism spectrum disorder) through direct reprogramming of the hypothalamus pituitary–adrenal axis in response to infection and by psychological stressors [19].

## Alteration of the gut microbiota

### Effect of antibiotics and other drugs on the microbiota

Because of the critical illness of admitted patients, antibiotics are frequently used in intensive care units [20]. Recent advances in understanding the role of intestinal microbiota spotlight potential harmful effects of these medications [21, 22] in that these therapies target pathogenic bacteria, but also the commensal ones making our microbiota [23] (Fig. 4). The impact of antibiotics is multifactorial: it depends on the antibiotic’s intrinsic characteristics (class, pharmacokinetic and pharmacodynamic properties) and the way in which it is used (e.g., dosage, duration, administration route) [24]. With its substantial biliary excretion (> 80%) and its activity against many anaerobic bacteria, clindamycin is a good example to illustrate the consequences of antibiotics on the microbiota. This drug alters bacterial diversity [25, 26] and favors the growth of intrinsically resistant microorganisms (e.g., *Clostridioides difficile*, *Enterococcus* spp. or Enterobacterales) making clindamycin a major risk factor for the development of *C. difficile* infection (CDI) [27]. In a similar way, macrolides [28], glycopeptides [29] or fluoroquinolones [30] have been shown to significantly modify the composition of the intestinal microbiota. Conversely, rifaximin (typically used in hepatic encephalopathy) seems to have a limited effect on microbial diversity while favoring the growth of beneficial bacteria [31–34]. Still in routine clinical settings, ranking the antibiotics according to their potential impact on the intestinal microbiota remains driven by expert opinions [35]. Indeed, while the impact of antibiotics has been extensively studied, the lack of standardization between studies hampers any type of comparison [36, 37]. In our opinion, an important point is that the antibiotic spectrum and the impact of the antibiotic on gut microbiota are not necessarily correlated [37]. To date, there is no hard data comparing the importance of microbiota dysbiosis between extended-spectrum antibiotics such as carbapenems and other broad-spectrum antibiotics [38, 39].

Bearing a special status because of its direct action on the microbiota, selective digestive decontamination (SDD) or selective oral decontamination (SOD) are also well-used in critically ill patients and can be cited in treatments modifying the microbiota [40]. Contrary to the patients not treated, the main variations in those treated with SDD are a decrease of *Enterobacteriaceae*, an increase of enterococci, and an impact on anaerobic bacteria [41]. Conversely, SOD seems to have a limited impact on the microbiota [40].

By analyzing the gut microbiomes of 1,135 Dutch patients exposed to various commonly used drugs, Zhernakova et al. showed that non-antibiotic drugs may also impact the gut microbiota [42]. Among them, proton pump inhibitors [43, 44], metformin [45], nonsteroidal anti-inflammatory drugs [46], or statins exerted a detectable effect on the composition of the gut microbiota. In addition to all cited molecules, many intensive care-specific therapies such as artificial feeding [47], mechanical ventilation, proton pump inhibitors [48], and vasopressors may also contribute to the microbiota dysregulation [3]. Among them, opioids, which also contribute to the slowing down of the intestinal transit, are widely used in ICU patients and possibly modulate the microbiota by increasing *Enterococcus* and *Staphylococcus* species and favoring their extra-intestinal dissemination as shown in a murine model of sepsis [49].

### Consequences of the alteration of the gut microbiota

MDRO are a growing burden in intensive care structures [50, 51]. An unaltered microbiota seems to be a key element in the fight against resistant organisms because of its ability to confront exogenous bacteria, including the resistant ones: this concept is called colonization resistance and protective organisms are beginning to be studied in greater detail [4, 52] (Fig. 4). A major study touching on the relation between the antibiotic use and the emergence of MDR bacteria was published by Donskey et al. [53] in the 2000s, showing that the intestinal concentrations of vancomycin-resistant *Enterococcus *spp. were correlated to the use of antibiotics with a marked activity on anaerobic bacteria, thereby altering the bacterial protective barrier and allowing the growth of resistant microorganisms. In animal models, *Clostridium scindens* was shown to slow the growth of *C. difficile* infection [54], *Blautia producta* and *Clostridium bolteae* that of vancomycin-resistant *Enterococcus* inhibition [55] and Lactobacilli, Clostridiales and anaerobes that of *Listeria monocytogenes* infection [56]. Several studies have analyzed and confirmed the increase of resistant bacteria after antibiotic administration, the latter altering colonization resistance, provoking, at the expense of the susceptible bacteria, a selection of MDR organisms, such as Enterobacterales [57, 58] (including extended-spectrum beta-lactamase-producing *E. coli* [59, 60]) or vancomycin-resistant *Enterococcus *spp. [59]*.* However, colonization with beta-lactamase-producing strains in high abundance may be also helpful: a recent animal study showed that a prior colonization by these strains may inactivate antibiotics in the gut after systemic treatment, protecting thus the microbiota from dysbiosis [61, 62]. Such observations have paved the way for the development of solutions aiming at protecting the microbiota [63, 64]. As a specific form of antibiotic therapy, SDD does not appear to increase the emergence of bacterial resistance in ICU [65], and seems to be paradoxically associated with a lower prevalence of rectal carriage of antibiotic-resistant Gram-negative bacteria in ICU [66, 67]. However, some observations have pointed to the SDD’s responsibility in the emergence of bacterial resistance after ICU stay [68]. A high relative abundance of resistant microorganisms in microbiota has an important clinical impact, because it favors their involvement in infections [36, 57, 60, 69], the duration of rectal carriage and shedding [70], insofar as high concentrations of MDRO in stools correlates with environmental contamination and may play an important role in MDRO transmission [71].

The microbiota is also a cornerstone for immunity development [72, 73] with mucosal gut immunity on the one hand and systemic immunity on the other hand. Indeed, many structures (e.g., Peyer’s patches) are involved in immune modulation for instance via immunoglobulin A (IgA) which constitutes a major gut immune component potentially targeting some gut microorganisms [74, 75]. The alteration of the gut microbiota may also lead to the dysregulation of the immune system [76]. A reduction of mucosal IgA concentration has been shown to be associated in mice with an increased abundance of gamma-Proteobacteria (which include Enterobacterales) associated with pro-inflammatory properties [77]. Similarly, an absence of IgA allows *Bacteroides thetaiotaomicron* to induce a pro-inflammatory state [78]. An alteration of the microbiota could also have consequences on the T cells and notably on T_H_-17 cells, which are involved in antimicrobial defense and have an action on intestinal epithelial cells, enabling production of antimicrobial peptides [79, 80]. The connection between dysbiosis and the immune system is illustrated by the increased susceptibility to asthma through dysregulation of T effector cells and IgE production in patients frequently exposed to antibiotics in their early infancy [81]. The interactions between gut bacteria and the host immune system can also occur via the production of specific bacterial metabolites [82]. SCFA concentration is associated with a reduced risk of colorectal adenoma and patients with inflammatory bowel disease present high levels of medium-chain fatty acids in feces comparatively to healthy subjects [83, 84]. Butyrate is also an energy source for the gut cells, and their shortage because of dysbiosis in critically ill patients may provoke immune dysregulation and cell death [85–87].

Alteration of microbiota and abnormalities of the immune system have consequences on the presence of bacteria with anti-inflammatory properties, creating a pro-inflammatory state in the guts of critically ill patients. At the species level, the decrease or even disappearance of some bacteria such as *Faecalibacterium prausnitzii* has been associated with the promotion of a pro-inflammatory state, as seen in other patients with inflammatory bowel diseases or digestive cancers [88–90]. Specifically in SARS-CoV-2 infection, a dysbiosis could be connected to the severity of COVID_19, with the drop of above-mentioned *Faecalibacterium prausnitzii* being associated with disease severity [91]. Furthermore, microbiota may be involved in certain types of inflammatory conditions [92]. Dysbiosis may alter the intestinal barrier, allowing systemic passage of bacterial components, metabolites or pathogen-associated molecular patterns (PAMPs) [93, 94], resulting in the production of pro-inflammatory mediators such as cytokines or chemokines [95]. This pathophysiological process is involved, for example in patients with type 2 diabetes via imidazole propionate, a microbial metabolite which contributes to insulin resistance [96]. Other inflammatory states may also be promoted by disturbances in the microbiota. The angiotensin converting enzyme 2 (ACE2) hydrolyses angiotensin II, which participates in pro-inflammatory events such as vascular permeability increase, or recruitment of infiltrating cells into the tissues [97]. In healthy patients, ACE2 is expressed in the small intestine, and the absence of this expression (e.g., malnutrition, gut injuries during critical illness), leads to an aberrant production of antimicrobial components induces modifications of the colon microbiota, favoring local inflammatory reactions, colitis, and even other organ dysfunctions [98]. Indeed, animal studies have shown the importance of the mesenteric lymph nodes in the gut-mediated lung injury and neutrophil activation, probably via toll-like receptor 4 stimulation or other pattern recognition receptors activation [99]. As an example, in the case of SARS-CoV-2 infection, specific modifications of the microbiota are correlated with a pro-inflammatory state: the presence of *Ruminococcus gnavus* and *Clostridium *spp. were correlated positively and negatively, respectively, with inflammatory markers [100]. Yeoh et al. [101] discretely corroborated these observations in finding associations between underrepresented gut bacteria with immunomodulatory properties and high concentrations of blood cytokines and biomarkers in severe patients; however, the small number of critically ill patients and the large proportion of patients who received antibiotics hinder the extrapolation of the results. Through analyses of viral transcriptional activity of fecal samples, those with a signature of high SARS-CoV-2 infectivity had higher abundances of bacteria with enhanced capacity for biosynthesis of nucleotide and amino acid, and carbohydrate metabolism. By contrast, fecal samples with a signature of low infectivity had higher abundances of short-chain fatty acids producing bacteria [102].

### Composition of the microbiota in ICU patients

A decade ago, Shimizu et al. [86] analyzed the gut microbiota of patients admitted in ICU with systemic inflammatory response syndrome (SIRS) criteria and highlighted that an altered microbiota in critically ill patients, and notably a decrease of obligate anaerobes and increase of pathogenic bacteria, was associated with an increased risk of infection or death. Further studies confirmed that the microbiota of ICU patients suffers from the emergence of low-diversity communities. Typically, Enterobacterales, *Staphylococcus *spp., *Enterococcus *spp. or yeasts such as *Candida albicans* classically involved in invasive diseases in critically ill patients, are taking over to the detriment of some species associated with a positive role in normal gut microbiota composition such as *Ruminococcus* spp., *Pseudobutyrivibrio* spp. or *Faecalibacterium prausnitzii* [34, 103, 104]. Recent studies have compared the composition of microbiota of several critically ill individuals and find some interpersonal variations, such as reduced diversity and richness [2, 105, 106].

### Association with infections and outcome

The prognosis of critically ill patients is determined, among many other things, by the occurrence of healthcare-associated infections, which may occur in up to 25% of critically ill patients during ICU stay, with significant consequences on survival or morbidity [107]. The rectal carriage of specific cultivable bacteria—especially Enterobacterales or *Enterococcus *spp.—detected at ICU admission seems to be associated with a higher rate of subsequent infection with the same micro-organism [69, 108, 109]. These observations are corroborated by a very recent study in ICU patients, which shows that throat or rectal carriage of extended-spectrum beta-lactamase producing Enterobacterales (ESBL-E) is a risk factor for developing ESBL-E ventilator-associated pneumonia [110]. Specifically in non-ventilated patients, the modification of oral and oropharyngeal microbiota with presence of *E. coli*, *P. aeruginosa* or *S. aureus* increase the risk of unspecific hospital-acquired pneumonia (HAP) [111]. Microbiota-associated considerations are also significant: altered fecal diversity is common in ICU patients and may also increase the rate of infections [86, 112]. First, in animal studies, gut microbiota seems to have a direct protective role against infections, notably pneumonia due to *S. pneumoniae* [113]. In ICU patients, ventilator-associated pneumonia (VAP) constitutes a common nosocomial complication. Gut bacteria seem to play a role in the pathogenesis of these VAP as they can colonize the oropharyngeal microbiota and then the respiratory tract, potentially leading to the development of an infection [114]. A recent study by Dickson et al. [115] showed that the gut microbiome influence the respiratory microbiome, and found an increased abundance of *Bacteroides *spp., an anaerobic bacteria from the gut in patients developing acute respiratory distress syndrome. Moreover, the most recent studies are beginning to show associations between VAP and the tracheal microbiota; in mechanically ventilated patients, the patients developing VAP seem to present a different microbiota compared to those who did not develop VAP [116]. As well as infections, altered gut microbiota seems to have an impact on the host’s outcome in critically ill situations. In animal studies, germ-free or antibiotic-treated mice (i.e., in which dysbiosis was induced) seem to be more susceptible to severe colitis [117]. In ICU patients, the use of SDD has been associated with a better patients’ outcome, notably in ICUs with low prevalence of antibiotic resistance [66, 67, 118, 119], but these observations have not been confirmed in ICUs with moderate-to-high prevalence of antibiotic resistance and when a parenteral cephalosporin was not associated [120]. A major study published by Freedberg et al. finally showed that a dominance of *Enterococcus *spp. at ICU admission is associated with short-term outcomes [69]. Recently, our group observed a strong correlation between the diversity of the intestinal microbiota of ICU patients and the relative abundance of *Enterococcus *spp., supporting that the quantification of *Enterococcus *spp. could be a potential biomarker for dysbiosis in that a high relative abundance seems to be associated with worse outcome [121]. Moreover, a recent study in patients who received allogeneic hematopoietic cell transplantation showed that the type of diet may influence the dysbiosis severity by modifying among others the abundance of *Enterococcus *spp. which causes GVHD [122].

## Perspectives: prevention and interventions (Table 2 and Fig. 4)

### Address the means to prevent the effect of antibiotics on the gut microbiota

Promising methods for preventing dysbiosis are emerging, with potential applications in ICU. Among them, use of beta-lactamase enzymes in patients treated by beta-lactams showed a preservational effect on microbiome integrity. Ribaxamase (developed by Synthetic Biologics and previously known as SYN-004) is a drug with the above-cited effect. Derived from the beta-lactamase of *Bacillus licheniformis* [63], it can hydrolyze residual ceftriaxone and other beta-lactam residues in the intestinal tract, with excellent tolerance and unchanged pharmacokinetics [64]; in animal studies, ribaxamase use prevented the alteration of microbial diversity after antibiotic administration. As an Ambler class B beta-lactamase, ribaxamase is not affected by beta-lactamase inhibitors such as tazobactam or sulbactam, in vivo [123]. After the publication of Phase I studies which have shown that ribaxamase was well tolerated [124], the first Phase II studies recently published seem to confirm that after administration of intravenous ceftriaxone, an adjuvant ribaxamase therapy lowered the risk of CDI [125]. Another innovative possibility for eliminating antibiotics from the digestive tract to avoid their effects is the use of an adsorbent nonspecific activated charcoal (being developed under the name of DaV-132 by the DaVolterra company), administered *per os* and neutralizing residual antibiotics in the colon, without altering the digestive absorption upward the ileocecal region. Already tested in a Phase I trial involving healthy subjects taking one-day treatment by amoxicillin [126], DaV-132 shows a high capacity of antibiotic absorbance (> 99%) in patients treated by a longer course of moxifloxacin [127]. More recently, this treatment shows signs for a potential decrease of mortality in *C. difficile*-infected animals [128]; studies targeting patients at high risk for *C. difficile* colitis are ongoing (ClinicalTrials.gov, Identifier: NCT03710694). As of today, this prevention of antibiotic-related dysbiosis is driven by antibiotic stewardship in that, whenever possible, the drug and posology with the lesser impact on the gut is preferred over others. Choosing the best antibiotic to avoid harmful impacts on patients’ microbiota must remain a major concern, specifically in ICU, because it has been shown that even a brief exposure to broad-spectrum antibiotics increases the risk of resistance emergence [129]. By extension, the antibiotic de-escalation from broad-spectrum antimicrobials to agents of a narrower spectrum or a lower ecological impact remains an important point to be considered in critically ill patients, as mentioned in international guidelines, even if there is no hard evidence if de-escalation has specific effects on bacterial resistance emergence [130]. To date, most of practitioners base their de-escalation on reducing the spectrum, which is not the only determinant of its impact on the intestinal microbiota [131]. For instance, imipenem has a very large spectrum of activity while its impact on the gut microbiota could not be demonstrated [132]. Precise data are still needed to include considerations related to the microbiota when determining the right antibiotics for patients [37].

**Table 2:** Advantages and disadvantages of potential interventions on the gut microbiota of intensive care patients

Fecal microbiota transplantation	Consists in the administration of fecal material from healthy individuals for restoring a normal microbiota Now recommended for the treatment of recurrent *C. difficile* infection Reasonably safe treatment (rare side-effects) May constitute an option for MDR bacteria eradication (still being explored) New indications need to be extensively explored: e.g., severe CDI, abundant diarrhea, adjuvant treatment in sepsis/multiorgan failure Only heterologous FMT may be considered in ICU Hard to implement as a routine practice in ICU Lack of evidence specifically in critically ill patients Numerous unanswered questions: selection of patients and donors, administration modalities (route, antibiotics management), storage
Ribaxamase (SYN-004)	Colon-delivered beta-lactamase hydrolyzing colonic beta-lactams residues Excellent tolerance Unchanged beta-lactams pharmacokinetics (observed for ceftriaxone) May prevent the alterations of microbial diversity after antibiotic administration
DaV-132	Adsorbent nonspecific activated charcoal with *per os* intake Neutralizes residual antibiotics in the colon and seems to have high capacity of antibiotic absorbance Ongoing studies targeting patients at high risk for *C. difficile* colitis (potential decrease of mortality in *C. difficile*-infected animals)
“Standard” probiotics	Living microorganisms used to prevent dysbiosis Antimicrobial properties, positive impact on immune system, reduced gut cell death Seems to reduce infections (especially VAP and *C. difficile* infections) and antibiotic consumption in critically ill patients Discordant mortality results Potential side-effects: sepsis, bacteremia, endocarditis, abscesses, VAP
SER-109	“Targeted” probiotics Bacterial spores from *Firmicutes* spp. which may reduce *C. difficile* proliferation

*MDR* multi-drug resistant, *CDI* *Clostridioides difficile* infection, *FMT* fecal microbiota transplantation, *ICU* intensive care unit, *VAP* ventilator-associated pneumonia

### Probiotics and related

Probiotics refer to living microorganisms whose aim is to provide a benefit. Recent animal studies showed that probiotics have many positive effects, including antimicrobial properties, positive impact on immune system or reduced gut cell death [133, 134]. In ICU, the use of these therapies was recently clarified after the publication by Manzanares et al. [135] of the largest meta-analysis of probiotics to date (2700 critically ill patients). Numerous probiotics were used in the trials included in this study: mainly *Saccharomyces boulardii*, *Lactobacillus* spp. and *Bifidobacterium* spp. Importantly, the use of probiotics was associated with decreased rate of infections [136] (especially VAP [137]) and decreased use of antibiotics, yet it was not associated with increased survival. *Lactobacillus plantarum* showed in this meta-analysis the most significant effect on the reduction of infections. A very recent large prospective, placebo-controlled trial, involving 2653 critically ill patients failed to demonstrate benefits of preventive administration of probiotics in these patients to avoid VAP [138]. However, these results need to be confirmed in other studies and notably probiotics should undergo precise safety evaluation: *Lactobacillus* spp. contained in probiotics have been involved in few cases of sepsis, pneumonias, abscesses or infectious endocarditis [139]. Preventive intake of probiotics seems to reduce incidence of *C. difficile*-associated diarrhea, and their side-effects [140, 141]; to date, probiotic prophylaxis cannot be recommended as mentioned in recent guidelines [142]. Moreover, probiotics have been associated with negative effects in other settings. In a RCT conducted by Besselink et al. in 2008, probiotics were associated with higher mortality in critically ill patients admitted for acute pancreatitis [143], thereby stressing that probiotics use would not be devoid of adverse effects, and they cannot be currently administrated to all critically ill patients.

Prebiotics, defined as extrinsic, specific substrates used by bacteria in order to modulate the microbiota composition, and synbiotics (combination of prebiotics and probiotics) are also potential options to control dysbiosis in critically ill patients. In the above-mentioned meta-analysis by Manzanares et al*.* [135], there were no difference between probiotics alone and synbiotics on the occurrence of infections. As addressed in a recent meta-analysis [144], synbiotic therapy and prebiotics may have a role in preventing events such as nosocomial infections. To date, RCTs evaluating synbiotics in critically ill patients show discordant results and failed to demonstrate a clear benefit of adjunctive synbiotic therapy on infections occurrence [145, 146].

A new generation of probiotics is now being developed. Among them, SER-109 is a hybrid mixture of probiotics in that it is made from a fecal transplant to which massive sporulation is triggered by alcohol. Phase Ib trials showed promising results for recurrent CDI [147] yet the results of the Phase 2 study were disappointing in this regard, likely due to underdosing of the product [148]. Phase III trials are ongoing (ClinicalTrials.gov, id: NCT03183128, NCT03183141). Other specific probiotics are also being developed, differing from available antibiotics in that they have been identified, selected, and carefully analyzed for specific properties demonstrated in vitro. Probiotics were studied in many other potential indications, but data are still lacking in the sepsis situations—maybe the more important because of antibiotic use—with only few studies with low sample size. As potential immune strengtheners, probiotics are wetting the appetite of researchers in SARS-CoV2 infections [149, 150], and start to be tested as an adjuvant treatment in few COVID-19 clinical trials (ClinicalTrials.gov, id: NCT04621071, NCT04390477, NCT04666116). In the same way, a clinical trial (ClinicalTrials.gov, id: NCT04251767) sought to evaluate the relevance of washed FMT in patients with COVID-19 but was unfortunately recently withdrawn; the use of FMT (e.g., from healthy donors or convalescent COVID-19 patients) as a specific experimental treatment for critically ill patients needs to be explored [151].

### Fecal microbiota transplantation

Fecal microbiota transplantation (FMT) is a procedure where feces from a healthy donor is introduced into the gastro-intestinal (GI) tract of a patient in order to reconstitute the intestinal microbiota and treat disorders associated with dysbiosis [152]. A renewed interest in this approach was signaled recently by the publication of the randomized controlled trial (RCT) by van Nood et al. [153] studying FMT in CDI, which is an undisputed model of a disorder directly connected to dysbiosis. A large body of evidence, including RCTs, systematic reviews and meta-analyses has now provided clear evidence that FMT is superior to antibiotics for curing patients with recurrent CDI (rCDI) with a clinical resolution of symptoms up to 90% of patients, and a significant reduction of the relapses [154]. Hence, FMT is now recommended as an option in the treatment of rCDI [153, 155, 156], and to date, no data supports the use of FMT in indications other than rCDI outside the context of research [157]. Because FMT stool includes practically all the bacteria, viruses, eukaryotes, and metabolites from the healthy donor, it potentially provides a more comprehensive approach to dysbiosis reversion and microbiota restoration, compared to the single or few bacterial strains included in probiotics [158, 159]. However, it introduces the risk of transmitting unwanted microorganisms (including pathogens) form the donor which could be detrimental to an already vulnerable host [152, 157]. Recently, DeFilipp et al. [160] described even two cases of transmission after FMT ESBL-*E. coli* bacteremia (one of these patients died). In order to reduce and prevent any adverse event related to the infused fecal material, potential donors have to undergo a medical interview and exhaustive testing in accordance with the European consensus conference on FMT in clinical practice [157]. The obtained transplant can be frozen, with no apparent loss of efficacy [161], and administered later in various ways: the upper and the lower route are classically described, but lyophilized or frozen pills are now preferred in the interest of patient compliance.

The capacity of FMT to eradicate the intestinal carriage of MDRO remains unclear, however. The first study to hypothesize that resistance to colonization with multidrug-resistant organisms (MDRO) could be increased by FMT compared stool samples of 20 patients with rCDI about to receive FMT, before and after the infusion, with the stool of the 3 donors. Before FMT, rCDI patients had a greater number and diversity of antibiotic resistance genes compared with donors and healthy controls. And after FMT, they observed a resolution of symptoms that correlated directly with a decreased number and diversity of antibiotic resistance genes, which was maintained in recipients for up to a year following FMT [162]. Nonetheless, the method used (metagenomic sequencing) lacks sensitivity and subdominant MDRO may have gone undetectable. Other small cohorts have reported a mixed effect of FMT on MDRO carriage. A single-center study [163] showed interesting results with a total eradication of MDR bacteria in 75 percent of the non-critically ill patients with blood disorders in their study. A recent meta-analysis suggested that FMT could be more effective for eradicating *Pseudomonas aeruginosa* than for *Klebsiella pneumoniae* [164]. Besides, a small RCT failed to demonstrate that 5 days of antibiotics followed by FMT could eradicate multidrug-resistant Enterobacterales [165].

In ICU, other indications for FMT could be considered such as an adjunctive treatment in sepsis and multiorgan failure [166]: few case reports have described FMT use in critically ill patients with septic shock of unclear cause with abundant diarrhea [158, 166–169], or in patients with antibiotic-associated diarrhea [170], with interesting results. However, performing FMT in ICUs comes with several challenges [171]. Like stem cells, autologous or heterologous FMT are two usable techniques; however, in ICU, only heterologous FMT seems to be feasible, given the fact that critically ill patients’ microbiota undergoes early changes, and it seems difficult to consider autologous FMT [171]. Moreover, difficult access to fecal transplants—for both techniques—requires the consideration of many factors for a routine implementation of FMT [152, 172]. Despite the existence of European guidelines, many questions remain unanswered about the use of FMT, specifically in critically ill patients [152, 157]: the necessary time-consuming physical examination and biological tests, the selection of patients and donors, the administration modalities, or the fecal transplant material storage. Technically, the transplantation at the patient’s bedside may face some difficulties for the administration modalities. In critically ill patients, the colonoscopy seems to be for now the preferred route for FMT, but other administration techniques such as through enteral feeding tube, lyophilized products [173], or oral capsules [174] need to be investigated in the coming years [175], given the fact that side-effects linked to the chosen administration route are rare (though a case of aspiration pneumonia after enteral FMT administration was reported) [176]. The timing of FMT also remains unknown, because it should be practiced during a period without antibiotic treatments [177, 178], which remains challenging in routine ICU practice.

## Conclusion

Monitoring the microbiota through next-generation sequencing and culture-based methods are a promising path to understanding how it connects to infection risk, inflammatory state, immune response, and outcome. As renal or hepatic markers used by intensivists to adapt therapeutic strategies, we need to build solid microbiota-associated markers designed to improve the care of critically ill patients. In a more proactive fashion, preserving or restoring the integrity of the gut microbiota remains challenging, but solutions are currently being developed. In conclusion, we believe that the role of the microbiota in the management of critically ill patients will grow in the next years.

## Data Availability

Not applicable.
